# Associations between Aquaglyceroporin Gene Polymorphisms and Risk of Stroke among Patients with Hypertension

**DOI:** 10.1155/2020/9358290

**Published:** 2020-03-25

**Authors:** Qingyun Tu, Li Yan, Chun Wang, Aohan Han, Yu Qin, Lan Cui, Quanyong Xiang

**Affiliations:** ^1^School of Public Health, Southeast University, Nanjing 210009, China; ^2^Department of Infection Prevention and Control, Zhongda Hospital of Southeast University, Nanjing 210009, China; ^3^Department of Medical Oncology, Thomas Jefferson University, Philadelphia, PA 19107, USA; ^4^Department of Noncommunicable Chronic Disease Control, Jiangsu Provincial Center for Disease Control and Prevention, Nanjing 210009, China

## Abstract

**Background:**

Dysregulations of *AQP7* and *AQP9* were found to be related to lipid metabolism abnormality, which had been proven to be one of the mechanisms of stroke. However, limited epidemiological studies explore the associations between *AQP7* and *AQP9* and the risk of stroke among patients with hypertension in China.

**Aims:**

We aimed to investigate the associations between genetic variants in *AQP7* and *AQP9* and the risk of stroke among patients with hypertension, as well as to explore gene-gene and gene-environment interactions.

**Methods:**

Baseline blood samples were drawn from 211 cases with stroke and 633 matched controls. Genomic DNA was extracted by a commercially available kit. Genotyping of 5 single nucleotide polymorphisms (SNPs) in *AQP7* (rs2989924, rs3758269, and rs2542743) and *AQP9* (rs57139208, rs16939881) was performed by the polymerase chain reaction assay with TaqMan probes.

**Results:**

Participants with the rs2989924 GG genotype were found to be with a 1.74-fold increased risk of stroke compared to those with the AA+AG genotype, and this association remained significant after adjustment for potential confounders (odds ratio (OR): 1.74, 95% confidence interval (CI): 1.23-2.46). The SNP rs3758269 CC+TT genotype was found to be with a 33% decreased risk of stroke after multivariate adjustment (OR: 0.67, 95% CI: 0.45-0.99) compared to the rs3758269 CC genotype. The significantly increased risk of stroke was prominent among males, patients aged 60 or above, and participants who were overweight and with a harbored genetic variant in SNP rs2989924. After adjusting potential confounders, the SNP rs3758269 CT+TT genotype was found to be significantly associated with a decreased risk of stroke compared to the CC genotype among participants younger than 60 years old or overweight. No statistically significant associations were observed between genotypes of rs2542743, rs57139208, or rs16939881 with the risk of stroke. Neither interactions nor linkage disequilibrium had been observed in this study.

**Conclusions:**

This study suggests that SNPs rs2989924 and rs3758269 are associated with the risk of stroke among patients with hypertension, while there were no statistically significant associations between rs2542743, rs57139208, and rs16939881 and the risk of stroke being observed.

## 1. Introduction

Stroke is the second most common cause of death worldwide and is the leading cause of long-term disability in developing and developed countries [1]. In China, there are more than one million people who die from stroke-related diseases per year. Evidence suggests that the pathogenesis of stroke is the result of interactions between genetic predispositions and environmental factors [2]. A large body of scientific research has indicated that gene polymorphisms modulate the pathophysiological processes of stroke and confer a small to moderate risk [3–5]. Studies in twins, families, and animal models provide substantial evidence for a genetic contribution to stroke [6, 7]. Recently, polymorphisms of genes involved in lipid metabolism have been found to be associated with stroke occurrence [8].

Aquaporins (AQPs) represent a family of transmembrane proteins permeable to water and, in some cases, also to other solutes [9]. Generally, the AQP family can be divided into three major subgroups based on their permeability characteristics and amino acid sequence homology [10]: AQP0, AQP1, AQP2, AQP4, and AQP5 belong to the group of classical aquaporins; AQP6, AQP8, AQP11, and AQP12 belong to the second subgroup called unorthodox aquaporins [11]; and *AQP3*, *AQP7*, *AQP9*, and *AQP10* belong to the third group, called aquaglyceroproteins, which are permeable to small uncharged molecules, such as urea, glycerol, or water [12–16].

In recent years, researchers pay more attention to the associations between AQPs' genetic polymorphisms and disease susceptibility. AQPs have been implicated in the regulation of both physiological and pathological water homeostasis and thus represent a promising target for alleviating stroke-induced cerebral edema [17]. Specifically, *AQP7*—the adipose-specific glycerol channel—represents a gateway for the delivery of adipose-derived glycerol into plasma [15, 18, 19] and it was identified as one of the dysregulated adipose tissue genes in obese humans [20]. *AQP7* is also known to play a pivotal role in glycerol metabolism in a wide range of tissues with implications for whole-body energy balance as well as the pathophysiology of obesity and development of insulin resistance [10]. Studies suggest that *AQP7* dysregulation may lead to an increased supply of glycerol for hepatic gluconeogenesis and to increased glucose levels in type 2 diabetes [11]. Moreover, *AQP9* is considered the main facilitator of glycerol uptake in hepatocytes [11, 21, 22]. Ribeiro et al. found that after focal transient ischemia in mice, a profound increase in *AQP9* immunolabeling was detected in astrocytes in peri-infarcted areas after 24 h of postocclusion, with a maximum at 7 days postocclusion [23]. Dysregulation of *AQP7* and *AQP9* may result in lipid metabolism abnormality, which is one of the important mechanisms of stroke.

However, epidemiological studies on the associations between *AQP7* and *AQP9* and the risk of stroke among patients with hypertension are still largely lacking. Therefore, this study is aimed at investigating the associations between *AQP7* and *AQP9* genetic variants and the risk of stroke among patients with hypertension.

## 2. Materials and Methods

### 2.1. Ethical Approval

This study was approved by the Institutional Review Board of Jiangsu Provincial Center for Disease Control and Prevention, and each participant signed a written informed consent for their participation.

### 2.2. Study Population

Participants in this study were recruited from the Follow-up Cohort Study of Hypertension (FCSH), which was established in 2010 in Jiangsu Province of China. In FCSH, over 12,000 patients with hypertension from 5 different counties (Jianye district in Nanjng, Peixian county in Xizhou, Haian county in Nantong, Lianshui county in Huaian, and Sihong county in Suqian) of Jiangsu Province were involved. Eligible criteria for inclusion were as follows: adults (18-70 years), the essential hypertension (with the systolic blood pressure (SBP) ≥ 140 mmHg and/or diastolic blood pressure (DBP) ≥ 90 mmHg), and blood pressure does not reach the control target (i.e., SBP ≥ 140 or DBP ≥ 90 mmHg). Patients were excluded if they met one of the following conditions: with secondary hypertension, having comorbidities (peripheral vascular diseases, coronary artery diseases, autoimmune diseases, systemic inflammatory diseases, blood diseases, malignant tumors, acute myocardial infarction, and stroke) in the previous 3 months of the study, or with life expectancy of less than one year. All participants were followed up once a year. At each follow-up visit, blood pressure and body weight were measured and information about lifestyle (e.g., smoking, drinking, and physical activity) and diseases (e.g., cardiocerebrovascular disease, cancers, and diabetes) was updated.

By the end of 2014, there were 301 person-times cases with stroke with confirmed diagnosis. The cumulative incidence of stroke was 2.51%, and the average follow-up time was 43.4 (±10.2) months. All patients with stroke were identified by at least two neurology specialists according to clinical signs and symptoms and/or imaging data. Among the 301 cases with stroke, 90 were excluded because of recurrent stroke (*N* = 38), no gene polymorphism test (*N* = 19), no qualified blood sample (*N* = 9), and having other severe diseases (such as cancer, severe emphysema, and severe liver and kidney function) (*N* = 24). We finally included 211 cases with stroke in this study.

The control group was composed of followed-up hypertensive patients who did not have stroke, deep vein thrombosis, or myocardial infarction. Cases and controls were matched on age (±3 years old), gender, baseline blood pressure (±5 mmHg), and residential area according to the ratio of 1 : 3. At last, 633 controls were included in this study.

### 2.3. Data Collection

All participants underwent a face-to-face interview by trained interviewers to complete a structured questionnaire, including demographic information, lifestyle information, medical history, and family history. After the interview, anthropometric indices (height, body weight, and waist circumference (WC)) and blood pressure were measured. Body weight and height were measured according to a standard protocol [24]. Body mass index (BMI) was calculated as weight in kilograms divided by height in meters squared (kg/m^2^). Blood pressure was measured using an automated device (OMRON Model HEM-7071, Omron Co.) at the nondominant arm 3 times consecutively with a 1~2 minute interval between two measurements while participants were in a seated position.

Blood samples were collected from participants after overnight fasting for at least 10 h. Serum total cholesterol (TC), low-density lipoprotein cholesterol (LDL-C), high-density lipoprotein cholesterol (HDL-C), triglycerides (TG), and fasting blood glucose (FBG) were measured using an autoanalyzer (Abbott Laboratories).

An individual was categorized as “overweight” if their BMI ≥ 25 kg/m^2^, and central obesity was defined according to the waist circumference (WC) of the participant as men > 90 cm or women > 80 cm [25]. A stringent quality assurance and quality control procedure was implemented to ensure the validity and reliability of study data.

### 2.4. SNP Selection and Genotyping Assay

We followed the methods of Wang et al. to select five single nucleotide polymorphisms (SNPs) (rs2989924, rs3758269, and rs62542743 in *AQP7* and rs57139208 and rs16939881 in *AQP9*) and assay genotyping [26]. Allelic discrimination was automatically completed using the Sequence Detection Systems Software version 2.3. The genotyping call rates for 5 SNPs were all >97% in this study. For quality control, the DNA samples from cases and controls and two blank controls were run in the same batch, and the operators performing detection and genotyping did not know the case or control status of the samples. Moreover, 10% of genotyping samples were randomly chosen to redo the assay blindly for consistency check.

### 2.5. Statistical Analysis

All the data in this study were double entered by a trained personnel using EpiData 3.5 and analyzed by SPSS 21.0. The differences in demographic characteristics and selected variables between cases and controls were compared using a Student *t*-test for continuous variables and a *χ*^2^ test for categorical variables. The Hardy-Weinberg equilibrium was tested by the goodness-of-fit *χ*^2^ test to compare the observed genotype frequencies to the expected ones among controls. The associations between genotypes and risk of stroke were evaluated using univariate and multivariate logistic regression analyses to determine the crude and adjusted odds ratios (OR) and their 95% confidence intervals (CI). All statistical tests were two sided. *P* < 0.05 was considered statistically significant. The genetic linkage balance test of 5 SNPs was performed using SNPstats software. The generalized multifactorial dimensionality reduction method (GMDR) was used to analyze the gene-gene interaction. Using the incidence of stroke at follow-up as the outcome variable, the five loci of genes rs16939881, rs57139208, rs62542743, rs3758269, and rs2989924 were named as SNP1, SNP2, SNP3, SNP4, and SNP5, respectively, in turn and were included in the GMDR model as the analysis factors. Meanwhile, the age, gender, duration of hypertension, family history of hypertension, family history of stroke, SBP, TC, TG, and FBG were included in the model as potential confounders. The haplotypes of SNPs on the same chromosome were analyzed by SNPstats software.

## 3. Results

### 3.1. Demographic Characteristics of Participants

No deviation from the Hardy-Weinberg equilibrium for the polymorphisms examined was observed in the genotype distributions between cases and controls. The demographic characteristics of participants are presented in Table 1. A total number of 211 patients with stroke and 633 controls were included in the study. Significant differences were observed between cases and controls in the duration of hypertension, family history of hypertension, or stroke. SBP, TC, TG, and FBG were higher among patients with stroke than those in their matched control group.

### 3.2. Associations between SNPs and the Risk of Stroke

The frequencies of genotype and allele of the SNP polymorphisms are shown in Table 2. The genotype distributions of AA, GG, and AG in rs2989924 of AQP7 were significantly different between cases and controls (*χ*^2^ = 8.260, *P* = 0.016). Participants with rs2989924 GG genotypes had a 1.55-fold increased risk of stroke (OR 1.55, 95% CI 1.12-2.15) compared to those with the AA+AG genotype, and this relationship remained significant after adjustment for covariates (age, gender, SBP, FBG, TC, TG, and LDL-C) (OR 1.74, 95% CI 1.23-2.46). When compared with the rs3758269 CC genotype, participants with CT+TT genotypes had a 33% decreased risk of stroke after adjustment for potential confounders (OR 0.67, 95% CI 0.45-0.99). No association was observed between the genotypes of rs62542743, rs57139208, and rs16939881 and the risk of stroke.

### 3.3. Stratified Analyses of the Associations between Genotypes of rs2989924 and rs3758269 and Risk of Stroke

Table 3 shows the results of stratified analyses. A significantly increased risk of stroke in the rs2989924 GG vs. AA+AG (recessive model) genotype was observed among men (OR 1.80, 95% CI 1.10-2.93) and the elderly (OR 1.81, 95% CI 1.20-2.75), and this association remained significant after adjustment for potential confounders (OR 2.23, 95% CI 1.32-3.77 and OR 2.20, 95% CI 1.41-3.44, respectively). Among the hypertensive population, the rs2989924 GG vs. AA+AG (recessive model) genotype was found to be associated with an increased risk of stroke among participants who were overweight or central obese, with an unadjusted OR of 2.35 (95% CI 1.45-3.81) and 1.81 (95% CI 1.22-2.70), respectively. However, among patients with ≥25 kg/m^2^ BMI, the rs3758269 CT genotype was associated with a reduced risk of stroke compared to those harboring the CC genotype (adjusted OR 0.40, 95% CI 0.21-0.75), and a similar result was observed in the CT+TT vs. CC genotype (dominant mode) (adjusted OR 0.43, 95% CI 0.24-0.77). The rs3758269 CT+TT genotype was associated with a reduced risk of stroke compared with the CC genotype among participants younger than 60 years old (adjusted OR 0.48, 95% CI 0.25-0.94).

### 3.4. Analysis of Interaction

Table 4 shows the gene-gene interactions, and the results suggested that there was no interaction.

### 3.5. Haplotype Analysis

The linkage disequilibrium (LD) test was conducted on rs62542743, rs3758269, rs2989924, rs57139208, and rs16939881. Results showed that there was a significant linkage imbalance between rs3758269 and rs2989924, and the *D*′ value is 0.9992. *D*′ < 0.75 between the other two SNPs indicated that there was no significant linkage imbalance. If only the SNPs with a frequency of more than 5% were included, three SNPs of *AQP7* can build 8 kinds of haplotype and two SNPs of *AQP9* can build 4 kinds of haplotype.  Table 5 shows the results after adjustment for age, gender, duration of hypertension, family history of hypertension, family history of stroke, SBP, TC, TG, and FBG. Individuals with the CTA haplotype in AQP7 have a higher risk of stroke compared to those with the highest frequency of single-body CCG (*P* < 0.05). As shown in Table 6, the analysis of AQP9 by SNPstats found that there was no statistically significant difference in the risk of stroke between individuals with the highest frequency of haplotype CC and those with the remaining haplotypes (*P* > 0.05).

### 3.6. The Logistic Analysis of 5 SNPs and Risk of Stroke

The five loci of genes rs2989924, rs3758269, rs62542743, rs57139208, and rs16939881; duration of hypertension; family history of hypertension; family history of stroke; SBP; TC; TG; FBG; and daily intake of fruits were used as independent variables, and stroke was used as a dependent variable for logistic regression analysis. The results of multivariate analysis are shown in Table 7, which indicated that high TC was a risk factor for stroke (OR 1.31, 95% CI 1.05-1.63) and the rs2889924 AG genotype may be a protective factor for stroke.

## 4. Discussion

In this population-based case-control study, we investigated 5 SNPs in the *AQP7* and *AQP9* genes and found that the SNPs of rs2989924 and rs3758269 in *AQP7* were associated with the risk of stroke, while no association was observed between *AQP9* SNPs and the risk of stroke.

This study found a significantly increased risk of stroke in rs2989924 GG vs. AA+AG (recessive model) genotypes among men. According to some preclinical and basic studies, female hormones like estrogen and progesterone may have the ability to reduce the incidence of stroke, cardiovascular disease, and the tissue damage after an occurrence of ischemia [27, 28]. In this study, we found that people aged 60 or above with the GG vs. AA+AG genotype variation in SNP rs2989924 were with an increased risk of stroke, while the CT+TT genotype variation in rs3758269 was found to be related to a reduced risk of stroke among people under 60 years old. Such findings indicated that aging might cause an adverse reverse genetic variation of the *AQP7* genotype and is likely to cause stroke and aggravate stroke symptoms [29]. Kim and Vemuganti's study had also reported that the tolerance of vasculature to blood pressure and the capacity of the brain's self-healing notably declined with increasing age, which contributed to increase the susceptibility of brain stroke [30].

Prudente et al. recruited 685 cases of type 2 diabetes mellitus (345 females/340 males) and 292 controls (185 females/107 males) in Caucasus and found that the risk of the AG+GG genotype was 1.36 times higher compared with the AA genotype (OR 1.36, 95% CI 1.01-1.84), while stratified analysis showed that the risk of the AG+GG genotype was 1.66 times higher in females who were obese than that of the AA genotype (OR 1.66, 95% CI 1.01-2.74) [31]. In this study, the finding that the *AQP7* rs2989924 genotype variation was a risk factor for obesity-related diseases is consistent with findings of Prudente et al. However, the rs3758269 CT genotype in patients with BMI (kg/m^2^) ≥ 25 can reduce the risk of stroke compared with the CC genotype and the CT+TT vs. CC genotype (dominant model) after adjustment for potential confounders (OR 0.40, 95% CI 0.21-0.75 and OR 0.43, 95% CI 0.24-0.77, respectively). According to the stratification analysis of WC, no association between the rs3758269 genotype and stroke was found. However, Asian women have a higher prevalence of abdominal and visceral adiposity than Caucasian women with the same BMI, indicating a significant correlation between WC and visceral adiposity volume [32]. Oikonomou et al. reported that the *AQP7* messenger RNA (mRNA) increased in younger obese prepubertal (YOP) children but decreased in the obese adolescents (OA) who also had increased insulin and homeostatic model assessment-insulin resistance (HOMA-IR) [33]. This may be due to the expression of *AQP7* in adipose tissue where it facilitates the efflux of glycerol, and *AQP7* deficiency has been linked to increased glycerol kinase activity and triglyceride accumulation in adipose tissue, leading to obesity and secondary development of insulin resistance [10]. No association between *AQP9* and the risk of stroke was found in this study, but *AQP9* was found to be the only aquaglyceroporin expressed in the brain and was detected in tanycytes [34]. In addition, *AQP9* and *AQP4* can act in synergy contributing to the facilitation of water movements between the CSF and brain parenchyma [35].

LD is the nonrandom association of alleles at different loci and plays an important role in diverse aspects of human genetics. In this study, we found that there was a strong linkage disequilibrium between rs3758269 and rs2989924 of the *AQP7* gene (*D*′ = 0.9992), while the LD relationships between the other loci were weak. By analyzing the LD association strength, the appropriate tagSNP is selected, which can help to reduce the number of SNPs in the study and find the SNP associated with the disease as a marker. A set of SNP combinations on the same chromosome that are interrelated and tend to be passed on to offspring as a whole is called haplotype. Haplotype association studies are more useful than single SNP analysis. In this study, *AQP7* and *AQP9* loci constitute haplotypes, and more than 5% of the haplotypes were included in the study. This study found that individuals carrying CTA haplotypes in *AQP7* had a higher risk of stroke than those with the highest frequency of CCG haplotypes (OR 1.56, 95% CI 1.05-2.32).

Multivariate logistic regression analysis was conducted to investigate the possible genetic and environmental factors associated with stroke, and results showed that higher TC was a risk factor of stroke (OR 1.31, 95% CI 1.05-1.63) and rs2889924 AG vs. GG (A>G) was a protective one. However, no interaction between *AQP7* and *AQP9* was found in this study.

This is the first study in China, which investigated the association between genetic variants in *AQP7* and *AQP9* and the risk of stroke among hypertensive patients. Nested with a large cohort study including more than 12,000 hypertensive cases with a >5-year follow-up, this case-control study included strictly matched cases and controls, so that results from this study are more likely to be reliable. In the future, further studies using large and independent samples are warranted to confirm the findings of this study. Moreover, future functional studies on associated SNPs will help to better understand the underlying biological mechanisms. To summarize, this study suggests that the genetic variants of *AQP7* SNPs are associated with the risk of stroke among patients with hypertension in China.

## Figures and Tables

**Table 1:** Demographic and clinical characteristics by cases with stroke and controls.

Variables	Cases with stroke *N* (%)	Controls *N* (%)	*P*
Degree of education			
Primary school and below	157 (74.4%)	436 (68.9%)	0.291
Junior high school	47 (22.3%)	167 (26.4%)	
High school and above	7 (3.3%)	30 (4.7%)	
Duration of hypertension (years)			
≤5	122 (60.7%)	416 (69.6%)	**0.020**
>5	79 (39.3%)	182 (30.4%)	
Familial history of hypertension	48 (23.1%)	102 (16.3%)	**0.029**
Familial history of stroke	10 (4.8%)	11 (1.7%)	**0.014**
Familial history of CHD	7 (3.4%)	11 (1.8%)	0.167
Familial history of diabetes	5 (2.4%)	11 (1.7%)	0.556
Central obesity	158 (74.9%)	480 (75.8%)	0.781
BMI (kg/m^2^)			
Normal	78 (37.0%)	243 (38.4%)	0.922
Overweight	89 (42.2%)	264 (41.7%)	
Obese	44 (20.9%)	126 (19.9%)	
SBP (mmHg)	153.64 ± 12.88^∗^	151.16 ± 10.60^∗^	**0.012**
DBP (mmHg)	94.87 ± 8.16^∗^	93.74 ± 6.92^∗^	0.071
TC (mmol/L)	4.49 ± 0.76^∗^	4.31 ± 0.79^∗^	**0.006**
TG (mmol/L)	1.90 ± 1.26^∗^	1.68 ± 1.02^∗^	**0.019**
FBG (mmol/L)	5.45 ± 2.31^∗^	5.07 ± 1.38^∗^	**0.023**
HDL-C (mmol/L)	1.63 ± 0.41^∗^	1.59 ± 0.39^∗^	0.130
LDL-C (mmol/L)	2.04 ± 0.77^∗^	2.01 ± 0.72^∗^	0.652

∗ represents mean ± SD. SD: standard deviation; CHD: coronary heart disease; BMI: body mass index; SBP: systolic blood pressure; DBP: diastolic blood pressure; TC: total cholesterol; TG: triglycerides; FBG: fasting blood glucose; HDL-C: high-density lipoprotein cholesterol; LDL-C: low-density lipoprotein cholesterol.

**Table 2:** Genotype frequency distribution in cases and controls.

Variable	Strokes, *N* (%)	Control, *N* (%)	*χ* ^2^	*P*	OR	95% CI	OR	95% CI^∗^
*rs2989924*								
AA	41 (19.5)	117 (19.0)	8.260	0.016	1.00			
AG	86 (41.0)	317 (51.4)			0.774	0.505-1.188	0.645	0.409-1.018
GG	83 (39.5)	183 (29.7)			1.294	0.833-2.010	1.292	0.817-2.043
AA	41 (19.5)	117 (19.0)	0.032	0.858	1.00			
AG+GG	169 (80.5)	500 (81.0)			0.965	0.649-1.433	0.885	0.585-1.340
AA+AG	127 (60.5)	434 (70.3)	6.987	0.008	1.00			
GG	83 (39.5)	183 (29.7)			**1.550**	**1.119-2.148**	**1.741**	**1.232-2.461**
*rs3758269*								
CC	164 (77.7)	456 (72.2)	2.958	0.228	1.00			
CT	44 (20.9)	159 (25.2)			0.769	0.527-1.124	0.687	0.456-1.035
TT	3 (1.4)	17 (2.7)			0.491	0.142-1.696	0.500	0.143-1.754
CC	164 (77.7)	456 (72.2)	2.525	0.112	1.00			
CT+TT	47 (22.3)	176 (27.8)			0.743	0.514-1.073	**0.669**	**0.450-0.994**
CC+CT	208 (98.6)	615 (97.3)	1.098	0.295	1.00			
TT	3 (1.4)	17 (2.7)			0.522	0.151-1.798	0.545	0.156-1.904
*rs62542743*								
CC	192 (91.0)	584 (92.3)	0.361	0.835	1.00			
CA	18 (8.5)	46 (7.3)			1.190	0.674-2.102	1.054	0.573-1.939
AA	1 (0.5)	3 (0.5)			1.014	0.105-9.804	0.935	0.093-9.371
CC	192 (91.0)	584 (92.3)	0.341	0.559	1.00			
CA+AA	19 (9.0)	49 (7.7)			1.179	0.678-2.053	1.047	0.579-1.892
CC+CA	210 (99.5)	630 (99.5)	0.000	1.000	1.00			
AA	1 (0.5)	3 (0.5)			1.000	0.103-9.665	0.931	0.093-9.325
*rs57139208*								
CC	163 (77.3)	491 (77.6)	0.980	0.613	1.00			
CT	46 (21.8)	130 (20.5)			1.066	0.729-1.559	1.068	0.717-1.591
TT	2 (0.9)	12 (1.9)			0.502	0.111-2.267	0.284	0.036-2.254
CC	163 (77.3)	491 (77.6)	0.009	0.924	1.00			
CT+TT	48 (22.7)	142 (22.4)			1.018	0.702-1.477	1.005	0.679-1.488
CC+CT	209 (99.1)	621 (98.1)	0.872	0.351	1.00			
TT	2 (0.9)	12 (1.9)			0.495	0.110-2.231	0.280	0.035-2.218
*rs16939881*								
CC	184 (87.2)	551 (87.0)	0.442	0.802	1.00			
CG	26 (12.3)	76 (12.0)			1.024	0.637-1.648	1.038	0.632-1.704
GG	1 (0.5)	6 (0.9)			0.499	0.060-4.173	0.554	0.065-4.686
CC	184 (87.2)	551 (87.0)	0.004	0.953	1.00			
CG+GG	27 (12.8)	82 (13.0)			0.986	0.619-1.571	1.002	0.617-1.628
CC+CG	210 (99.5)	627 (99.1)	0.432	0.511	1.00			
GG	1 (0.5)	6 (0.9)			0.498	0.060-4.157	0.551	0.065-4.661

OR: odds ratio; CI: confidence interval. ^∗^Adjusted for age, gender, systolic blood pressure, fasting glucose, total cholesterol, triglycerides, and low-density lipoprotein cholesterol.

**Table 3:** Stratified analysis between the *AQP7* gene and the risk of stroke.

Variable	(Control/stroke)				OR (95% CI)	OR^∗^ (95% CI)	OR (95% CI)	OR^∗^ (95% CI)	OR (95% CI)	OR^∗^ (95% CI)	OR (95% CI)	OR^∗^ (95% CI)
rs2989924	AA	AG	GG	AA	AG		GG		AA+AG		GG vs. AA+AG	
Males	58/18	122/41	68/40	1.00	1.08 (0.57-2.05)	0.71 (0.36-1.43)	1.90 (0.98-3.66)	1.79 (0.90-3.56)	1.37 (0.76-2.48)	1.11 (0.60-2.07)	**1.80 (1.10-2.93)**	**2.23 (1.32-3.77)**
Females	59/23	195/45	115/43	1.00	0.59 (0.33-1.06)	0.57 (0.31-1.07)	0.96 (0.53-1.74)	0.99 (0.53-1.85)	0.73 (0.43-1.25)	0.73 (0.41-1.30)	1.40 (0.90-2.17)	1.46 (0.91-2.32)
Age ≤ 60 (years)	52/19	131/40	69/27	1.00	0.84 (0.44-1.58)	0.61 (0.30-1.24)	1.07 (0.54-2.13)	0.86 (0.41-1.80)	0.92 (0.51-1.66)	0.71 (0.37-1.35)	1.21 (0.71-2.07)	1.19 (0.66-2.13)
>60 (years)	65/22	186/46	114/56	1.00	0.73 (0.41-1.31)	0.64 (0.35-1.18)	1.45 (0.81-2.59)	1.62 (0.89-2.95)	1.01 (0.59-1.71)	0.10 (0.58-1.73)	**1.81 (1.20-2.75)**	**2.20 (1.41-3.44)**
BMI (kg/m^2^) < 25	54/18	168/47	103/33	1.00	0.84 (0.45-1.57)	0.79 (0.40-1.56)	0.96 (0.50-1.86)	1.04 (0.51-2.11)	—	0.89 (0.47-1.67)	—	1.23 (0.73-2.07)
≥25	63/23	149/39	80/50	1.00	0.72 (0.40-1.30)	0.58 (0.31-1.11)	1.71 (0.95-3.10)	1.67 (0.89-3.11)	1.07 (0.62-1.82)	0.97 (0.55-1.71)	**2.14 (1.36-3.36)**	**2.35 (1.45-3.81)**
Without central obesity	33/10	73/23	45/20	1.00	1.04 (0.45-2.43)	0.84 (0.34-2.08)	1.47 (0.61-3.54)	1.23 (0.48-3.15)	1.20 (0.55-2.65)	0.99 (0.43-2.29)	1.43 (0.74-2.75)	1.39 (0.68-2.85)
With central obesity	84/31	244/63	138/63	1.00	0.70 (0.43-1.15)	0.62 (0.37-1.05)	1.24 (0.74-2.06)	1.30 (0.77-2.21)	0.89 (0.57-1.41)	0.87 (0.54-1.41)	**1.59 (1.09-2.32)**	**1.81 (1.22-2.70)**
rs3758269	CC	CT	TT	CC	CT		TT		CC+CT		TT vs. CC+CT	
Males	178/76	63/23	10/0	1.00	0.86 (0.49-1.48)	0.80 (0.44-1.46)	—	—	0.74 (0.43-1.27)	0.70 (0.39-1.26)	—	—
Females	278/88	96/21	7/3	1.00	0.69 (0.41-1.17)	0.59 (0.33-1.07)	1.35 (0.34-5.35)	1.32 (0.33-5.32)	0.74 (0.44-1.22)	0.65 (0.37-1.13)	1.47 (0.37-5.78)	1.47 (0.37-5.90)
Age ≤ 60 (years)	177/68	68/19	10/0	1.00	0.73 (0.41-1.30)	0.54 (0.27-1.07)	—	—	0.63 (0.36-1.13)	0.48 (0.25-0.94)	—	—
>60(years)	279/96	91/25	7/3	1.00	0.80 (0.48-1.32)	0.78 (0.46-1.33)	1.25 (0.32-4.91)	1.25 (0.31-5.03)	0.83 (0.51-1.34)	0.82 (0.49-1.36)	1.31 (0.33-5.15)	1.32 (0.33-5.31)
BMI (kg/m^2^) < 25	249/71	79/28	6/0	1.00	1.24 (0.75-2.06)	1.06 (0.61-1.85)	—	—	—	0.98 (0.57-1.70)	—	—
≥25	207/93	80/16	11/3	1.00	0.45 (0.25-0.80)	0.40 (0.21-0.75)	0.61 (0.17-2.23)	0.65 (0.17-2.45)	0.47 (0.27-0.81)	0.43 (0.24-0.77)	0.72 (0.20-2.62)	0.79 (0.21-2.99)
Without central obesity	112/42	36/10	5/1	1.00	0.74 (0.34-1.62)	0.75 (0.32-1.77)	0.53 (0.06-4.70)	0.68 (0.07-6.19)	0.72 (0.34-1.52)	0.74 (0.33-1.68)	0.57 (0.07-4.99)	0.72 (0.08-6.51)
With central obesity	344/122	123/34	12/2	1.00	0.78 (0.51-1.20)	0.67 (0.42-1.07)	0.47 (0.10-2.13)	0.46 (0.10-2.13)	0.75 (0.49-1.45)	0.65 (0.41-1.03)	0.50 (0.11-2.25)	0.51 (0.11-2.34)

OR: odds ratio; CI: confidence interval. ^∗^Adjusted for age, gender, systolic blood pressure, fasting glucose, total cholesterol, triglycerides, low-density lipoprotein cholesterol, course of hypertension, family history of hypertension, and family history of stroke.

**Table 4:** Gene interaction analysis by GMDR method.

Model combination	Training sample accuracy	Verify sample accuracy	Cross-validation consistency	*P*
SNP3 SNP5	0.567	0.527	4/10	0.1719
SNP1 SNP4 SNP5	0.578	0.542	4/10	0.1719
SNP1 SNP2 SNP4 SNP5	0.592	0.536	6/10	0.1719
SNP1 SNP2 SNP3 SNP4 SNP5	0.608	0.542	10/10	0.1719

**Table 5:** Association between haplotypes of three SNPs of the *AQP7* gene and the risk of stroke.

Haplotype	rs62542743	rs3758269	rs2989924	Frequency	OR (95% CI)	*P*
Haplo.base	C	C	G	0.5520	1.00	—
Haplo.2	C	C	A	0.2804	1.12 (0.86-1.47)	0.430
Haplo.3	C	T	A	0.1243	**1.56 (1.05-2.32)**	**0.029**

**Table 6:** Association between haplotypes of two SNPs of the *AQP9* gene and the risk of stroke.

Haplotype	rs62542743	rs16939881	Frequency	OR (95% CI)	*P*
Haplo.base	C	C	0.8308	1.00	—
Haplo.2	T	C	0.1004	1.05 (0.72-1.53)	0.80
Haplo.base	C	C	0.8308	1.00	—

**Table 7:** The logistic analysis of 5 SNPs and the risk of stroke.

Variables	OR	95% CI	*P*
rs2889924AG vs. GG	0.513	0.353-0.746	<0.001
AA vs.GG	0.782	0.496-1.232	0.782
Higher TC	1.307	1.047-1.631	0.018
Fasting blood glucose	1.144	1.037-1.263	0.007
Systolic blood pressure	1.015	1.001-1.029	0.041

## Data Availability

The data used to support the findings of this study are available from the corresponding author upon reasonable request.
